# Metformin-associated lactic acidosis and temporary ileostomy: a case report

**DOI:** 10.1186/1752-1947-8-449

**Published:** 2014-12-20

**Authors:** Carla Margiani, Luigi Zorcolo, Paolo Mura, Marcello Saba, Angelo Restivo, Francesco Scintu

**Affiliations:** 26 Department of General Surgery, University Colorectal Unit, Cittadella Universitaria, SS 554, bivio per Sestu – 09042, Monserrato, Cagliari, Italy; 27 Department of Anesthesia and Intensive Care, University of Cagliari, Cagliari, Italy

**Keywords:** Ileostomy, Lactic acidosis, Metformin

## Abstract

**Introduction:**

Lactic acidosis is a well-known complication of the anti-hyperglycemic biguanide agent metformin, especially in peculiar but not rare clinical conditions. Attempts to reduce the incidence of this adverse reaction have been enforced by national agencies over the years. The Italian Medicines Agency recently released a safety recommendation on prescribing the drug and with regard to the existence of several conditions that contraindicate drug continuation, such as dehydration, infection, hypotension, surgery or hyperosmolar contrast agent infusion, but the recommendation does not mention the increased risk related to stoma. The present case report is, to our knowledge, the first in the literature of metformin-associated lactic acidosis in a patient with a recently created ileostomy and low anterior resection for rectal cancer.

**Case presentation:**

A 70-year-old Caucasian man who had undergone low anterior resection with total mesorectal excision and temporary loop ileostomy creation at our institution returned to our department 30 days later because of nausea, vomiting, diffuse abdominal pain and anuria of about 24 hours’ duration. During his physical examination, the patient appeared dehydrated and had tachypnea and a reduced level of consciousness. His laboratory tests showed that he had acute kidney injury and severe lactic acidosis.

**Conclusion:**

An ileostomy puts patients at high risk for output losses that can lead to dehydration and electrolyte abnormalities. The assessment of the losses through the stoma, especially the ileostomy, should be added to the recommendations issued by pharmacovigilance societies. The present clinical case illustrates the need for clinicians on surgical wards to carefully evaluate patients before resuming metformin therapy and to provide appropriate information at discharge to patients with type 2 diabetes mellitus who have undergone ileostomy. Furthermore, this case report highlights the increasing need for more training of general physicians regarding both surgical and internal medicine problems that may arise in the post-operative course after major surgery in patients with co-morbidities.

## Introduction

Metformin is the recommended first-line therapy for most patients with type 2 diabetes mellitus (T2DM) [1]. Despite its being generally safe and associated with a reduction of cardiovascular events, diabetes-related endpoints and overall mortality [2], metformin may lead to a rare but extremely serious adverse reaction: metformin-associated lactic acidosis (MALA). MALA is defined as a high anion gap metabolic acidosis with high circulating lactate levels and without hypoperfusion (type B described by Cohen and Woods) [3]. It is associated with a mortality rate up to 45% [4].

On the basis of a suspected increase in the incidence of MALA [5], the Italian Medicines Agency (AIFA) recently released and publicized a safety recommendation for assessment of renal function in patients for whom the drug is prescribed and suspension of therapy in cases of dehydration, infection and hypotension, as well as before surgery or diagnostic procedures involving hyperosmolar contrast agent infusion [5].

A temporary ileostomy, which is often created to protect low colorectal anastomoses after radical surgery for colorectal cancer (CRC), is a relatively simple procedure and is usually well tolerated by the patient. However, it may cause loss of fluids and electrolytes, and this can be a life-threatening condition in a patient taking metformin.

In this context, we report a case of a patient with severe MALA that developed after creation of a temporary loop ileostomy. To the best of our knowledge, no similar cases have been described in the literature.

## Case presentation

A 70-year-old Caucasian man (weight: 75kg; height: 165cm) with T2DM, hypertension and prostatic hypertrophy was referred to our unit for CRC treatment. His usual medications included metformin (3000mg/day), irbesartan/hydrochlorothiazide 150mg/12.5mg/day and tamsulosin (0.4mg/day).

Ten weeks after receiving pre-operative chemoradiotherapy, the patient underwent a low anterior rectal resection with a diverting ileostomy. Metformin treatment was suspended 3 days before the operation.

The patient’s post-operative course was uneventful. His renal function was normal; his blood glucose was between 150mg/dl and 200mg/dl after a meal; and his stoma output was approximately 600ml/day.

Metformin therapy was resumed 5 days after surgery, when the patient returned to a normal diet. He was discharged to home in good condition on the ninth post-operative day.

Two weeks later, he returned to our department because of nausea, vomiting and diffuse abdominal pain. In the preceding days, he had noticed an increased stoma output that required him to empty the stoma bag four or five times per day. He also referred to being anuric for the preceding 24 hours.

During his examination, the patient appeared dehydrated and had tachypnea and a reduced level of consciousness. His blood pressure was 90/60mmHg, and his pulse rate was 90/min. His laboratory tests showed acute kidney injury (AKI) (creatinine, 8.94mg/dl; blood urea nitrogen, 324mg/dl) with severe lactic acidosis (pH, 6.99; partial pressure of carbon dioxide, 12mmHg; base excess, -26.9 mM).

Aggressive hydration with crystalloids and intravenous bicarbonate infusion was initiated. The patient’s internal jugular vein was cannulated, and he was promptly transferred to the hemodialysis unit. Hemodialysis was performed for 6 hours and repeated 12 hours later for 3 hours using bicarbonate-buffered dialysate. His chemical profile and clinical condition then improved dramatically. His acidosis resolved over the course of 24 hours; his spontaneous diuresis resumed after 48 hours; and his renal function recovered. The patient was discharged 8 days after admission with a serum creatinine level of 2.5mg/dl and a blood urea nitrogen concentration of 50mg/dl. His ileostomy was closed about 1 month later, and he did not experience any further similar episodes. At a follow-up examination almost 1 year later, he was well and disease-free.

## Discussion

The incidence of CRC in older patients is increasing, resulting in a greater number of patients who present with concomitant medical illnesses [6]. In a meta-analysis published in 2005 [7], Larsson and colleagues showed a relationship between T2DM and an increased risk of CRC in both women and men (relative risk: 1.30, 95% confidence interval: 1.20 to 1.40). Further studies have shown that diabetes is associated with increased risk of CRC mortality [8, 9].

Because of the increasing incidence of its risk factors, the global prevalence of T2DM is predicted to reach 5.4% (300 million people) by 2025 [10], and adverse reactions to drugs commonly prescribed for this condition can be anticipated to increase accordingly.

MALA is a rare condition. In the largest case series published in the literature to date, the authors reported no more than dozens of episodes over years of observation [3]. Nevertheless, taken together, all of the case series point out the constant association of MALA with predisposing factors that in various ways affect metformin clearance or energy metabolism, such as altered renal function, dehydration, other drug interactions, congestive heart failure, hepatic failure, respiratory failure and drug overdose [3]. A common feature in different case series is that the prognostic effect of drug accumulation is less important than the seriousness of the underlying medical condition.

Our present case report is, to the best of our knowledge, the first in the literature of MALA in a patient with a recently created ileostomy and low anterior resection for CRC. After ileostomy creation, output usually increases until the third or fourth day post-operatively. The period between the fourth and sixth days post-surgery are characterized by stabilizing output. A 7-week period of steady decrease in volume and thickening of output ensues [11, 12]. Dehydration and electrolyte derangements most frequently occur in the early post-operative period. In a recent study, dehydration was found to be the most frequent indication for hospital readmission after diverting ileostomy creation [13]. In our patient, it is likely that ileostomy output remained high after discharge without an oral intake adequate to offset losses. The patient was also receiving diuretics, which certainly contributed to a condition of severe dehydration and electrolyte depletion. Furthermore, the patient continued to take metformin despite having become unwell. Dehydration led to pre-renal AKI, which was likely worsened by the concomitant consumption of irbesartan, resulting in accumulation of the drug, hypotension and consequent severe lactic acidosis.

## Conclusions

Safety recommendations released by the AIFA in July 2011 dictate metformin contraindications and recommend assessing renal function when the drug is prescribed. They also highlight the existence of conditions that contraindicate drug continuation, such as dehydration, infections, hypotension, surgery or hyperosmolar contrast agent infusion, but they do not mention the increased risk related to a stoma. Surgery poses special challenges to patients with diabetes because of the stress response and interruption of food intake. Although ileostomy cannot be considered a direct cause of MALA, it is certainly a high-risk condition for output losses that often lead to dehydration, electrolyte abnormalities, vitamin deficiencies and malnutrition. For these reasons, the assessment of the losses through the stoma should be added to the recommendations issued by pharmacovigilance societies. In this setting, ordinary recommendations regarding ongoing medical therapies (anti-hypertensive and diuretic treatment in our patient) should be followed with particular attention to eventual discontinuation of drugs that put the patient at risk for dehydration or AKI.

The case that we report illustrates the need for clinicians on surgical wards to take special care in using metformin to treat patients with T2DM who have ileostomies. A careful evaluation is advisable before the resumption of metformin therapy in these patients, and appropriate information should be given to the patient at discharge. Another important aspect that emerges from our case report is the increasingly urgent need for more training of general physicians regarding both surgical and internal medicine problems that may arise in the post-operative course after major surgery in patients with comorbidities.

## Consent

Written informed consent was obtained from the patient for publication of this case report. A copy of the written consent is available for review by the Editor-in-Chief of this journal.

## Abbreviations

AIFA: Italian Medicines Agency
AKI: Acute kidney injury
CRC: Colorectal cancer
MALA: Metformin-associated lactic acidosis
T2DM: Type 2 diabetes mellitus.

## References

[1] Prospective UK, Diabetes Study (UKPDS) Group: **Effect of intensive blood-glucose control with metformin on complications in overweight patients with type 2 diabetes (UKPDS 34).** *Lancet* 1998, **352:**854–865. A published erratum appears in *Lancet* 1998, **352:**1558 9742977

[2] Cohen RD, Woods HF: **Metformin and lactic acidosis.** *Diabetes Care* 1999, **22:**1010–1011. 10.2337/diacare.22.6.1010 10.2337/diacare.22.6.1010 10372265

[3] Lalau JD, Race JM: **Lactic acidosis in metformin-treated patients: prognostic value of arterial lactate levels and plasma metformin concentrations.** *Drug Saf* 1999, **20:**377–384. 10.2165/00002018-199920040-00006 10.2165/00002018-199920040-00006 10230584

[4] Renda F, Mura P, Finco G, Ferrazin F, Pani L, Landoni G: **Metformin-associated lactic acidosis requiring hospitalization: a national 10 year survey and a systematic literature review.** *Eur Rev Med Pharmacol Sci* 2013,**17**(Suppl 1)**:**45–49. 23436666

[5] World Health Organization, International Agency for Research on Cancer (WHO/IARC): **Higher blood vitamin D levels are associated with significantly decreased colon cancer risk in European populations.** Press release no. 198 (22 January 2010). [http://www.iarc.fr/en/media-centre/pr/2010/pdfs/pr198_E.pdf] (accessed 7 November 2014).

[6] Janssen-Heijnen ML, Houterman S, Lemmens VE, Louwman MW, Maas HA, Coebergh JW: **Prognostic impact of increasing age and co-morbidity in cancer patients: a population-based approach.** *Crit Rev Oncol Hematol* 2005, **55:**231–240. 10.1016/j.critrevonc.2005.04.008 10.1016/j.critrevonc.2005.04.008 15979890

[7] Larsson SC, Orsini N, Wolk A: **Diabetes mellitus and risk of colorectal cancer: a meta-analysis.** *J Natl Cancer Inst* 2005, **97:**1679–1687. 10.1093/jnci/dji375 10.1093/jnci/dji375 16288121

[8] Yang YX, Hennessy S, Lewis JD: **Insulin therapy and colorectal cancer risk among type 2 diabetes mellitus patients.** *Gastroenterology* 2004, **127:**1044–1050. 10.1053/j.gastro.2004.07.011 10.1053/j.gastro.2004.07.011 15480982

[9] Vigneri P, Frasca F, Sciacca L, Pandini G, Vigneri R: **Diabetes and cancer.** *Endocr Relat Cancer* 2009, **16:**1103–1123. 10.1677/ERC-09-0087 10.1677/ERC-09-0087 19620249

[10] Miller RA, Birnbaum MJ: **An energetic tale of AMPK-independent effects of metformin.** *J Clin Invest* 2010, **120:**2267–2270. 10.1172/JCI43661 10.1172/JCI43661 PMC2898617 20577046

[11] Tang CL, Yunos A, Leong AP, Seow-Choen F, Goh HS: **Ileostomy output in the early postoperative period.** *Br J Surg* 1995, **82:**607. 10.1002/bjs.1800820510 10.1002/bjs.1800820510 7613926

[12] Soybel DI: **Adaptation to ileal diversion.** *Surgery* 2001, **129:**123–127. 10.1067/msy.2001.107980 10.1067/msy.2001.107980 11174703

[13] Messaris E, Sehgal R, Deiling S, Koltun WA, Stewart D, McKenna K, Poritz LS: **Dehydration is the most common indication for readmission after diverting ileostomy creation.** *Dis Colon Rectum* 2012, **55:**175–180. 10.1097/DCR.0b013e31823d0ec5 10.1097/DCR.0b013e31823d0ec5 22228161

